# Effect of probiotic supplementation on gastrointestinal motility, inflammation, motor, non-motor symptoms and mental health in Parkinson’s disease: a meta-analysis of randomized controlled trials

**DOI:** 10.1186/s13099-023-00536-1

**Published:** 2023-03-06

**Authors:** Jong Mi Park, Sang Chul Lee, Chorom Ham, Yong Wook Kim

**Affiliations:** Department and Research Institute of Rehabilitation Medicine, Yonsei University College of Medicine, 50-1 Yonsei-Ro, Seodaemun-Gu, Seoul, 03722 Republic of Korea

**Keywords:** Parkinson’s disease, Probiotics, Gastrointestinal motility, Inflammation, Meta-analysis

## Abstract

**Background:**

Parkinson’s disease (PD) is the second most common neurodegenerative disease worldwide. Gut dysbiosis is hypothesized to cause PD; therefore, whether probiotics can be used as adjuvants in the treatment of PD is being actively investigated.

**Aims:**

We performed a systematic review and meta-analysis to evaluate the effectiveness of probiotic therapy in PD patients.

**Methods:**

PUBMED/MEDLINE, EMBASE, Cochrane, Scopus, PsycINFO and Web of Science databases were searched till February 20, 2023. The meta-analysis used a random effects model and the effect size was calculated as mean difference or standardized mean difference. We assessed the quality of the evidence using the Grade of Recommendations Assessment, Development and Evaluation (GRADE) approach.

**Results:**

Eleven studies involving 840 participants were included in the final analysis. This meta-analysis showed high-quality evidence of improvement in Unified PD Rating Scale Part III motor scale (standardized mean difference [95% confidence interval]) (− 0.65 [− 1.11 to − 0.19]), non-motor symptom (− 0.81 [− 1.12 to − 0.51]), and depression scale (− 0.70 [− 0.93 to -0.46]). Moderate to low quality evidence of significant improvement was observed in gastrointestinal motility (0.83 [0.45–1.10]), quality of life (− 1.02 [− 1.66 to − 0.37]), anxiety scale (− 0.72 [− 1.10 to − 0.35]), serum inflammatory markers (− 5.98 [− 9.20 to − 2.75]), and diabetes risk (− 3.46 [− 4.72 to − 2.20]). However, there were no significant improvements in Bristol Stool Scale scores, constipation, antioxidant capacity, and risk of dyslipidemia. In a subgroup analysis, probiotic capsules improved gastrointestinal motility compared to fermented milk.

**Conclusion:**

Probiotic supplements may be suitable for improving the motor and non-motor symptoms of PD and reducing depression. Further research is warranted to determine the mechanism of action of probiotics and to determine the optimal treatment protocol.

**Supplementary Information:**

The online version contains supplementary material available at 10.1186/s13099-023-00536-1.

## Introduction

Parkinson’s disease (PD) is the second most common neurodegenerative disease after Alzheimer’s disease. According to the Global Burden of Disease study, the incident number of PD in 2019 was 1081.72 × 10^3^, which is an increase of 159.73% since 1990 [1]. The incidence of PD is increasing globally with the aging population, and has become a challenge to global health. PD is characterized by motor symptoms, such as resting tremors, rigidity, bradykinesia, and postural instability, as well as by non-motor symptoms, such as gastrointestinal dysfunction, urinary incontinence, sweating, drooling, and neuropsychiatric problems [2]. Consequently, these features lower the quality of life of patients with PD. Recently, the role of the gut–brain axis—a bidirectional connection between the enteric and central nervous systems—has garnered attention [3, 4]. “Gut dysbiosis” refers to a change in immunity, inflammation, and neuromodulation caused by microorganisms in the gastrointestinal tract and is considered to play a role in the pathophysiology of PD. Currently, active research on this topic is ongoing [5–7]. Changes in the compositions of important microbes are thought to affect behavior, neurotransmitter synthesis, microglial function, neurogenesis, and the blood–brain barrier; thus, these changes are ultimately implicated in the pathophysiology of PD [4].

According to the World Health Organization, probiotics are defined as “living microorganisms that, when administered in adequate amounts, provide a health benefit to the host” [8]. Given the anti-inflammatory effects of probiotics, they may be an adjuvant treatment option for the management of PD. However, previous clinical results have demonstrated that the effectiveness of probiotics in patients with PD is variable [9–16]. This meta-analysis aimed to analyze the quantitative effects of probiotics on gastrointestinal symptoms, inflammation, metabolic disease risk, and PD symptoms in patients with PD.

## Methods

This meta-analysis study was conducted in accordance with the Preferred Reporting Items for Systematic Reviews and Meta-Analyses (PRISMA) 2020 statement [17, 18]. The PRISMA checklist is provided in Additional file 1. This review was registered in the International Prospective Register of Systematic Reviews (registration number CRD42022356798) on September 9, 2022.

### Search strategy

Relevant studies were systematically searched for in the PUBMED/MEDLINE, EMBASE, Cochrane, Scopus, and PsycINFO databases from inception until December 12, 2022 and we updated our search on February 20 2023. Additional articles were obtained by searching for citations in the list of included research and review articles. The search strategy was as follows: (“probiotic” OR “yeast” OR “yogurt” OR “fermented product” OR “lactobacillus” OR “bifidobacterium” OR “fermented dairy product” OR “symbiotics” OR “cultured milk products”) AND (“Parkinson” OR “Parkinson’s disease” OR “parkinsonism”) (Additional file 2).

### Inclusion and exclusion criteria

The study inclusion criteria followed the Population, Intervention, Comparison, Outcomes and Study design (PICOS) framework. The target population included adult patients with idiopathic Parkinson's disease who were 18 years old or older and diagnosed according to certain criteria (PD UK Social Brain Bank Standard, Queen Square Brain Bank Standard). The consumption/administration of probiotics such as pills or fermented milk over a period of time was considered an “intervention.” For comparison, those in whom probiotics were not administered or in whom a placebo was administered comprised the control group. The included outcomes were: (1) gastrointestinal symptoms with bowel movement, stool type, and constipation symptom data; (2) inflammation with data relating to inflammatory markers (nitric oxide, malondialdehyde, and high-sensitivity C-reactive protein) and antioxidant markers (total glutathione level and antioxidant capacity); (3) risk of metabolic syndrome as determined by fasting plasma glucose, serum insulin, and serum cholesterol levels; and (4) scores of Parkinson's disease-related scales: The Parkinson's Disease Questionnaire (PDQ-39), Non-Motor Symptoms Questionnaire (NMSQ), and Unified Parkinson's Disease Rating Scale (UPDRS). The study design included only randomized controlled trials that reported baseline and post-intervention data or changes in baseline data. Non-human studies, cohort studies, case reports, and studies that did not adhere to the PICOS framework were excluded.

### Data extraction and quality assessment

Two reviewers (JMP and YWK) independently extracted relevant information from studies, including the name of the first author, year of publication, patient demographic data, type of intervention (probiotic strain), intervention protocol, mean age, sex (male, %), sample size, and outcomes.

To identify the risk of bias, the quality of the included studies was evaluated using the Revised Cochrane Risk of Bias Tool for Randomized Trials (RoB2) to identify the risk of bias. The RoB2 assesses the following five components of risk of bias: the randomization process, deviations from the intended interventions, missing outcome data, measurement of the outcome, and selection of the reported results. Each part was evaluated as having a low risk of bias, some concerns of bias, high risk of bias, or no information [19]. Discrepancies in data extraction and quality assessment were resolved through discussion between the investigators. Following the recommendations of previous studies [20, 21], we also planned to evaluate publication bias via funnel plots once more than three studies were reviewed.

### Grading of the evidence

The Grading of Recommendations, Assessment, Development, and Evaluation (GRADE) tool was used to assess the certainty of the evidence of the included meta-analyses, consisting of five domains: (1) risk of bias, (2) inconsistency, (3) indirectness, (4) imprecision, and (5) publication bias. The certainty of the evidence was rated as high, moderate, low, or very low [22].

### Statistical analysis

Cohen's kappa statistic was used to calculate the level of agreement between the two reviewers regarding study inclusion. In this meta-analysis, we performed statistical standardization of the effect sizes of probiotics on Parkinson’s. First, the mean and standard deviation (SD) of the change-from-baseline values in the treatment and control groups were calculated. For studies with insufficient data, the mean and SD values of the changes from baseline values were calculated with reference to Chapter 6 of the Cochrane Handbook (version 6.3). In the trials that examined the different effects of probiotics, participants in each category were included in a separate meta-analysis. The effect sizes were expressed as standard mean differences (SMD) in a random effects model, and 95% confidence intervals (CI) between the treatment and control groups were calculated for each study and measure. Due to the relatively small number of heterogeneous studies, we used a random-effects meta-analysis with Hartung–Knapp–Sidik–Jonkman (HKSJ) adjustments [23]. Heterogeneity between studies was assessed using I^2^ and *P*-values from the Cochrane Q test. Publication bias was assessed by visual inspection of funnel plots and the Egger bias test. All statistical tests were two-sided, and *P* < 0.05 was considered statistically significant using Stata version 17 (Stata Corp LP, College Station, TX, USA).

## Results

### Study identification and characteristics

**Fig. 1:**
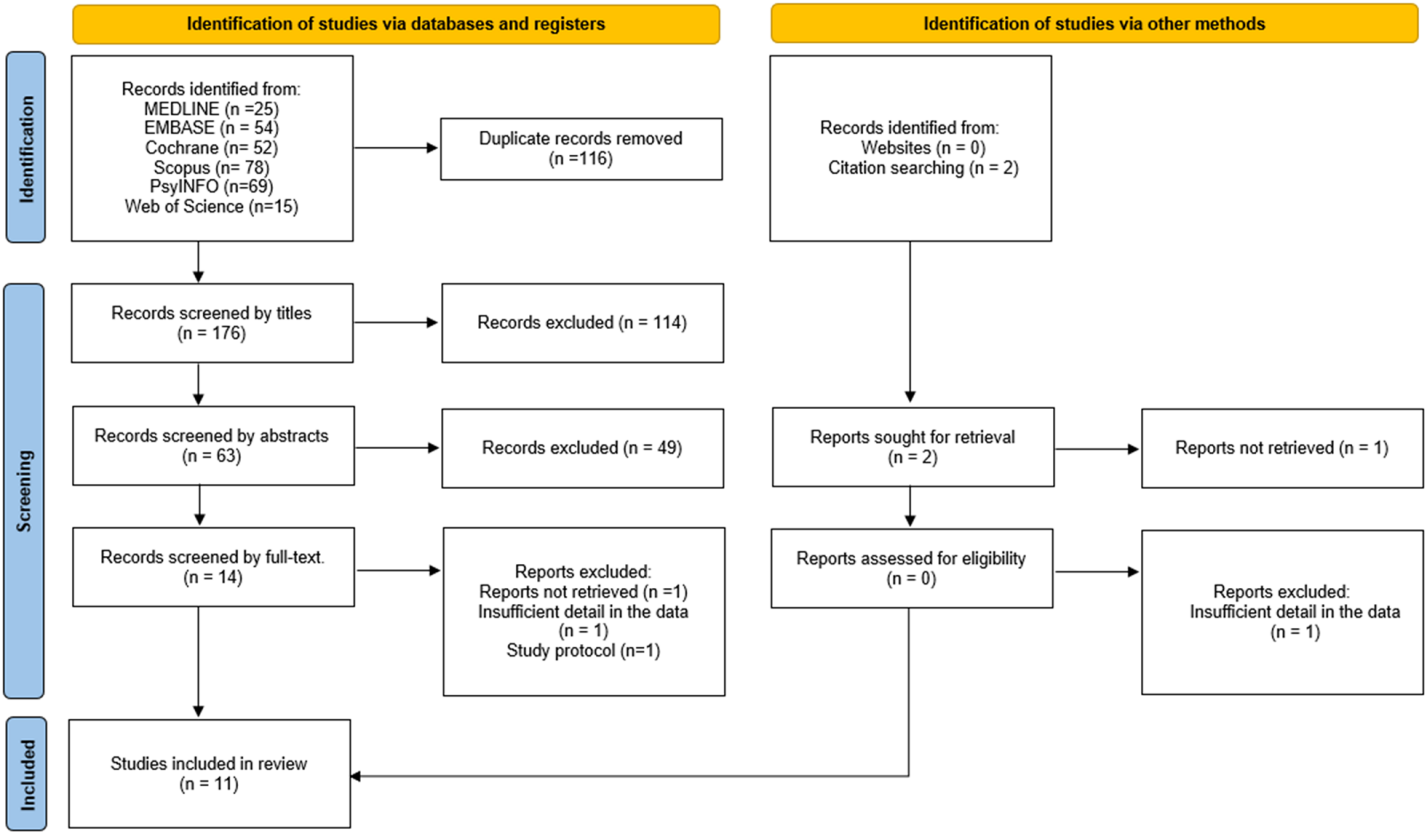
PRISMA flow-chart of the screening and selecting processes of the studies

A total of 293 studies were screened, of which 116 duplicate studies were excluded. After reading the titles and abstracts, 114 and 49 papers were excluded, respectively. Of the remaining 14 studies, three were excluded for various reasons (reports not retrieved, insufficient detail in the data, and incomplete study protocol information), and 11 were finally included in the meta-analysis. The degree of agreement of the full-text review phase was calculated using the Kappa score (kappa = 0.831; standard error = 0.160), which showed good agreement among the reviewers. A flowchart depicting the selection of studies is presented in Fig. 1. This meta-analysis included 11 studies and 840 patients with PD.

The number of participants in the included studies ranged from 40 [11] to 122 [16] participants, and the average age of the participants was between 66.5 [24] and 75.6 [11] years. All studies included both men and women. The duration of probiotic administration ranged from 4 [9, 13, 15] to 12 [10, 11, 14, 16, 10–11] weeks. Probiotics were administered in the form of fermented milk [9, 13, 16], capsules [10–12, 10–12, 10–12] or powder in sachets [26]. The detailed characteristics of the included studies are summarized in Table 1.

**Table 1:** Characteristics of the included studies

Study	Study design	No. of participants (experimental/control)	Age, years^a^ (experimental/control groups)	Probiotics	Control group	Frequency and duration	Outcome measure	Main finding
Barichella et al. [9]	Randomized double-blind clinical trial	80/40	71.8 ± 7.7/69.5 ± 10.3	Fermented milk containing multiple probiotic strains and prebiotic fiber	Pasteurized, fermented, fiber-free milk	Once daily for 4 weeks	1. Bowel movements 2. Bristol Stool Scale score 3. Treatment satisfaction 4. Treatment continuation	Probiotics were superior to placebo in improving constipation in patients with PD
Borzabadi et al. [10]	Randomized double-blind clinical trial	25/25	66.7 ± 10.7/ 66.9 ± 7.0	Capsule 8 × 10^9^ CFU *Lactobacillus acidophilus* *Bifidobacterium bifidum* *L. reuteri* *Lactobacillus fermentum* (2 × 10^9^ CFU each)	Placebo capsule	Once daily for 12 weeks	1. Gene expression (*IL-1, IL-8, TNF-α, TGF-β, PPAR-γ, VEGF*, and *LDLR*) 2. Biomarkers of oxidative stress (total glutathione and nitric oxide)	The probiotics group had significantly improved gene expression levels of *IL-1, IL-8, TNF-α, TGF-β*, and *PPAR-*γ; however, the gene expression of *VEGF* and *LDLR* and the number of biomarkers of inflammation and oxidative stress were unaffected
Du et al. [25]	Randomized clinical trial	23/23	68.39 ± 7.55/66.65 ± 8.66	Capsule 5 × 10^9^ CFU *Bacillus licheniformis* 1 × 10^7^ CFU *Lactobacillus acidophilus, Bifidobacterium longum,* *Enterococcus faecalis*	None	Three times daily for 12 weeks	1. Bowel movements 2. Bristol Stool Scale scores 3. Constipation (PAC-SYM) 4. Quality of life (PAC-QOL)	The probiotics group demonstrated improved bowel movements and improved scores on symptom scales associated with constipation (PAC-SYM scores and PAC-QOL scores)
Georgescu et al. [11]	Randomized controlled clinical trial	20/20	75.6 ± 9.7/69.8 ± 5.6	Capsule 60 mg *Lactobacillus acidophilus Bifidobacterium infantis*	Trimebutine 200 mg three times daily	Twice daily for 12 weeks	1. Abdominal pain 2. Bloating 3. Constipation	Probiotics improved abdominal pain and bloating similar to trimebutine; however, trimebutine was more effective for constipation with incomplete evacuation
Ibrahim et al. [12]	Randomized double-blind clinical trial	22/26	69.0 ± 5.2/70.5 ± 6.1	Capsule 3 × 10^10^ CFU *Lactobacillus* sp. *Bifidobacterium* sp. fructo-oligosaccharides lactose	Fermented milk	Twice daily for 8 weeks	1. Garrigues Questionnaire 2. Gut transit time 3. Quality of life (PDQ39-SI) 4. Motor symptoms (MDS-UPDRS) 5. Non-motor symptoms (NMSQ)	Probiotics led to improved bowel opening frequencies and whole gut transit times in PD
Michela et al. [13]	Randomized double-blind clinical trial	80/40	N/R/N/R	Fermented milk containing multiple probiotic strains and prebiotic fiber	Pasteurized, fermented, fiber-free milk	Once daily for 4 weeks	1. Complete bowel movements	Probiotics were superior to placebo in improving numbers of complete bowel movements
Mehrabani et al. [26]	Randomized double-blind clinical trial	40/40	68.2 ± 7.7/69.1 ± 8.2	Powder in sachets 5 × 10^9^ CFU *Lactobacillus acidophilus* *Lactobacillus rhamnosus Lactobacillus plantarum Bifidobacterium longum Streptococcus* thermophilus	Maltodextrin	Once daily for 12 weeks	1. Biomarkers of oxidative stress (TAC, TOS, OSI, GSH, MDA) 2. Quality of life (PDQ39) 3. Mental status (Hospital Anxiety and Depression Scale) 4. Fatigue Severity Scale scores	The probiotics group had increased TAC, reduced MDA levels, and reduced OSI values, and it also had lower levels of depression and displayed improvements in well-being and cognitive impairment
Sun et al. [24]	Randomized double-blind clinical trial	48/34	66.46 ± 6.98/68.76 ± 6.91	Capsule 3 × 10^10^ CFU *Bifidobacterium animalis subsp. lactis*	Maltodextrin	Once daily for 12 weeks	1. Number of spontaneous defecation incidents 2. Bristol Stool Scale scores 3. Motor symptoms (MDS-UPDRS) 4. Quality of life (PAC-QOL) 5. Hamilton Anxiety Rating Scale scores 6. Hamilton Depression Scale scores	Probiotics improved sleep quality and alleviated anxiety and gastrointestinal symptoms
Tamtaji, O. R., et al. (2019)[14]	Randomized double-blind clinical trial	30/30	68.2 ± 7.8/67.7 ± 10.2	Capsule 8 × 10^9^ CFU *Lactobacillus acidophilus* *Bifidobacterium bifidum* *Lactobacillus reuteri* *Lactobacillus fermentum* (2 × 10^9^ CFU each)	Placebo capsule	Once daily for 12 weeks	1. Motor symptoms (MDS-UPDRS) 2. High-sensitivity CRP 3. Biomarkers of oxidative stress (total glutathione, malondialdehyde) 4. Diabetes markers (fasting plasma glucose, Insulin, Insulin sensitivity check index) 5. Dyslipidemia markers (triglycerides, VLDL, LDL, HDL)	Probiotics had useful impacts on MDS-UPDRS scores and some metabolic profiles
Tan et al. [15]	Randomized double-blind clinical trial	34/38	70.9 ± 6.6/68.6 ± 6.7	Capsule 10^9^ CFU *Lactobacillus Acidophilus* *Lactobacillus reuteri* *Lactobacillus gasseri* *Lactobacillus rhamnosus* *Bifidobacterium bifidum Bifidobacterium longum Enterococcus faecalis* *Enterococcus faecium*	Placebo capsule	Once daily for 4 weeks	1. Bowel movements per week 2. Average stool consistency 3. Quality of life (PAC-QOL) 4. Constipation severity scores	Probiotics were effective for relieving constipation in patients with PD
Yang et al. [16]	Randomized double-blind clinical trial	63/59	N/R/N/R	Fermented milk containing 10^9^ CFU *Lactobacillus casei* strain	Placebo capsule	Once daily for 12 weeks	1. Quality of life (PDQ39) 2. Non-motor symptoms (NMSQ) 3. Hamilton Anxiety Rating Scale scores 4. Hamilton Depression Rating Scale scores	Probiotics may be a useful approach in managing the non-motor symptoms of PD

N/R, not recorded; CFU, colony-forming units; IL, interleukin; TNF, tumor necrosis factor; TGF, transforming growth factor; PPAR, peroxisome proliferator-activated receptor; VEFG, vascular endothelial growth factor; LDLR, low-density lipoprotein receptor; PAC-SYM, Patient Assessment of Constipation Symptoms; PAC-QOL, Patient Assessment of Constipation Quality of Life, UPDRS, Unified Parkinson's Disease Rating Scale; NMSQ, Non-Motor Symptoms Questionnaire; PDQ-39, Parkinson's Disease Questionnaire; PDSS, Parkinson’s Disease Sleep Scale; TAC, total antioxidant capacity; TOS, total oxidant status; OSI, oxidative stress index; GSH, glutathione; MDA, malondialdehyde; CRP, C-reactive protein; VLDL, very-low-density lipoprotein; LDL, low-density lipoprotein; HDL, high-density lipoprotein

^a^Mean age ± standard deviation

### Risk of bias assessment and assessment of publication bias

All 11 studies were randomized trials, and the majority (n = 7) described the randomization methods employed. However, only the abstract was available for two [13, 16] studies, which did not provide sufficient information, while two other studies [11, 25] did not sufficiently explain the allocation concealment method employed. The types of probiotics utilized in the intervention and placebo groups were identical and blinded in most studies; therefore, the risk of bias due to deviations from the intended intervention was low. However, two studies [16, 25] did not comment on the details of the placebo intervention protocol. A missing outcome bias was also reported in one study [16], and this was low risk in seven studies. Although most studies did not mention detection biases, gastrointestinal symptoms, inflammation, metabolic disease risk, and PD scores were measured quantitatively using various scales. All studies were rated as having a low-risk bias in outcome measures as the influence of knowledge was small.

**Fig. 2:**
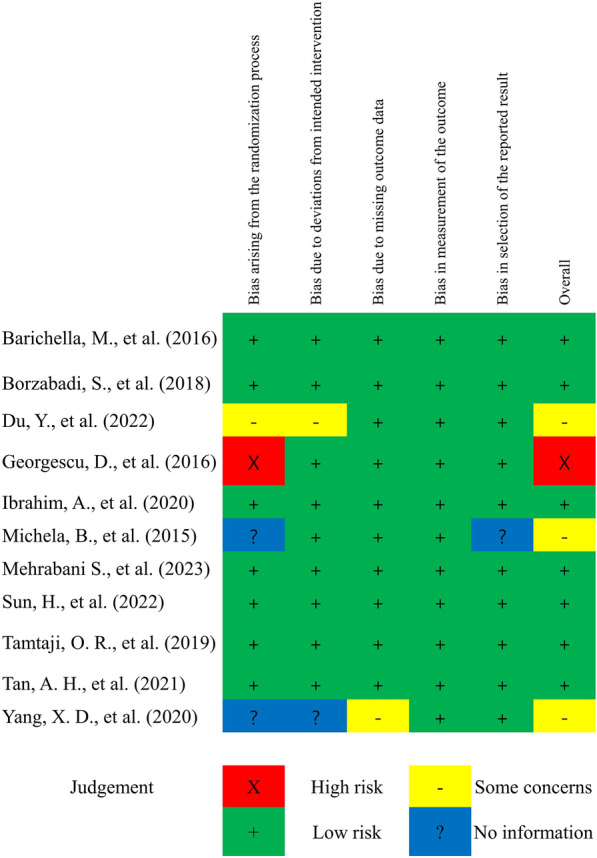
Risk of bias of the included studies. The risk of bias was assessed using the Cochrane Risk of Bias 2.0 Tool

All studies except one [13] were conducted according to a pre-randomized study protocol; therefore, the reported results were rated as having low selection bias. A traffic light plot for the assessment of each included study is shown in Fig. 2. The publication bias for each meta-analysis is shown in Additional file 3 as a funnel plot. In addition, publication bias was confirmed using Egger's test. Statistically significant publication biases were identified for gastrointestinal motility (*P* = 0.02), inflammation markers (*P* = 0.02), diabetes risk (*P* = 0.001), dyslipidemia risk (*P* = 0.01), and subgroup analyses (*P* = 0.01). Factors such as Bristol Stool Scale scores (*P* = 0.82), constipation symptom reduction levels (*P* = 0.16), antioxidant marker levels (*P* = 0.05), UPDRS Part III scores (*P* = 0.19), quality of life measures (*P* = 0.23), anxiety scale scores (*P* = 0.62), and depression scale scores (*P* = 0.62) showed no significant publication biases.

### Effects of probiotics on gastrointestinal symptoms

**Fig. 3:**
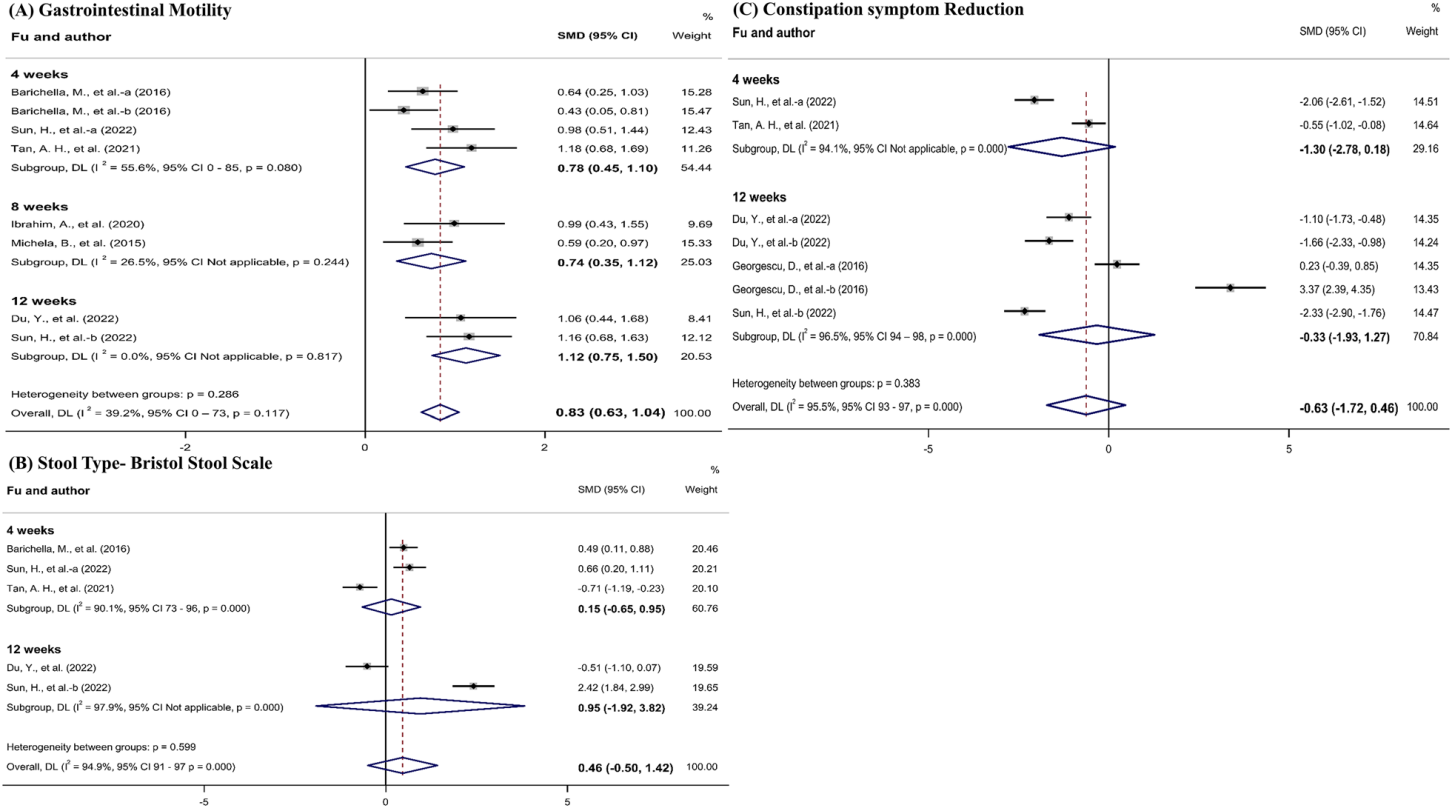
Meta-analysis of the effects of probiotics on gastrointestinal symptoms in Parkinson’s disease.

Gastrointestinal motility per week were reported in six studies [9, 12, 13, 15, 12–13] that included eight comparisons. The meta-analysis showed significant improvement in the probiotics groups (SMD, 0.83; 95% CI 0.63 to 1.04). Subgroup analyzes were performed according to the follow-up period, as the length of follow-up varied between studies and ranged from 4 to 12 weeks. Three studies [9, 15, 24] that followed up after 4 weeks showed significant improvement in the probiotics groups (SMD, 0.78; 95% CI 0.35 to 1.12). Two studies [12, 13] that followed up after 8 weeks showed significant improvement in the probiotics groups (SMD, 0.74; 95% CI 0.35 to 1.12). Two studies [24, 25] that followed up after 12 weeks showed significant improvement in the probiotics groups (SMD, 1.12; 95% CI 0.75 to 1.50) (Fig. 3A). The gastrointestinal motility quality of evidence was estimated as moderate performing the GRADE system (based on the publication bias) (Table 2).

**Table 2:** Summary of the findings and quality of evidence assessment using the GRADE approach

Outcome measure	Summary of findings	Quality of evidence assessment (GRADE)						
	No. of patients (trials)	Effect size (95% CI)	Risk of bias^a^	Inconsistency^b^	Indirectness^c^	Imprecision^d^	Publication bias^e^	Quality of evidence^f^
Gastrointestinal motility	210 (8)	0.83 (0.63, 1.04)	Not serious	Not serious	Not serious	Not serious	Serious	Moderate
Stool type: Bristol Stool Scale	210 (5)	0.46 (− 0.50, 1.42)	Not serious	Serious	Not serious	Serious	Not serious	Low
Constipation symptom reduction	258 (7)	− 0.63 (− 1.72, 0.46)	Serious	Serious	Serious	Serious	Not serious	Very low
Inflammation markers	250 (4)	− 5.98 (− 9.20, − 2.75)	Not serious	Serious	Serious	Not serious	Serious	Very low
Antioxidant markers	330 (5)	0.92 (− 0.28, 2.13)	Not serious	Serious	Serious	Serious	Not Serious	Very low
Diabetes risk	240 (4)	− 3.46 (− 4.72, − 2.20)	Not serious	Serious	Serious	Not Serious	Serious	Very low
Dyslipidemia risk	240 (5)	− 1.18 (− 2.48, 0.12)	Not serious	Serious	Serious	Serious	Serious	Very low
UPDRS Part III	219 (3)	− 0.65 (− 1.11, − 0.19)	Not serious	Not serious	Not serious	Not serious	not serious	High
NMSQ	177 (2)	− 0.81 (− 1.12, − 0.51)	Not serious	Not serious	Not serious	Not serious	Not serious	High
Quality of life	509 (7)	− 1.02 (− 1.66, − 0.37)	Not serious	Serious	Not serious	Not serious	Not serious	Moderate
Anxiety Scale	366 (4)	− 0.72 (− 1.10, − 0.35)	Not serious	Serious	Not serious	Not serious	Not serious	Moderate
Depression Scale	366 (4)	− 0.70 (− 0.93, − 0.46)	Not serious	Not Serious	Not serious	Not serious	Not serious	High
Gastrointestinal motility: fermented milk	360 (3)	0.55 (0.33, 0.77)	Not serious	Not serious	Not serious	Not serious	Serious	Moderate
Gastrointestinal motility: capsules	392 (6)	1.06 (0.85, 1.28)	Not serious	Not serious	Not serious	Not serious	Serious	Moderate

UPDRS, Unified Parkinson's Disease Rating Scale; NMSQ, Non-Motor Symptoms Questionnaire.

^a^Risk of bias based on the Revised Cochrane Risk of Bias Tool for Randomized Trials (RoB2)

^b^Downgraded if a significant and unexplained heterogeneity (I^2^ > 50%, *P* < 0.10) was not explained by meta-regression or subgroup analysis results

^c^Downgraded if there were any factors related to the participants, interventions, or results that limited the generalizability of the results

^d^Downgraded if the 95% confidence interval (95% CI) crossed the benefit-or-harm boundary

^e^Downgraded if there was evidence of publication bias using Egger's test

^f^Because all included studies were meta-analyses of randomized clinical trials, the certainty of the evidence was graded as high for all outcomes by default and then downgraded according to prespecified criteria. Quality was graded as high, medium, low, or very low.

Bristol stool scale were reported in four studies included five comparisons. The meta-analysis showed no significant difference between the experimental and control groups (SMD, 0.46; 95% CI − 0.50 to 1.42). Three studies [9, 15, 24] that followed up after 4 weeks showed no significant difference (SMD, 0.15; 95% CI − 0.65 to 0.95). Two studies [24, 25] that followed up after 12 weeks showed no significant difference (SMD, 0.95; 95% CI − 1.92 to 3.82) (Fig. 3B). The bristol stool scale quality of evidence was estimated as low performing the GRADE system (based on the inconsistency and imprecision) (Table 2).

Symptoms of constipation were reported in four studies [11, 15, 24, 25] that included seven comparisons. There were no significant differences in the summary effect size between the groups (SMD, − 0.63; 95% CI − 1.72 to 0.46). Two studies [15, 24] that followed up after 4 weeks showed no significant difference (SMD, − 1.30; 95% CI − 2.78 to 0.18). Three studies [11, 24, 25] that followed up after 12 weeks showed no significant difference (SMD, − 0.33; 95% CI − 1.93 to 1.27) (Fig. 3C). The constipation symptom quality of evidence was estimated as very low performing the GRADE system (based on the risk of bias, inconsistency, indirectness and imprecision) (Table 2).

### Effects of probiotics on inflammation

**Fig. 4:**
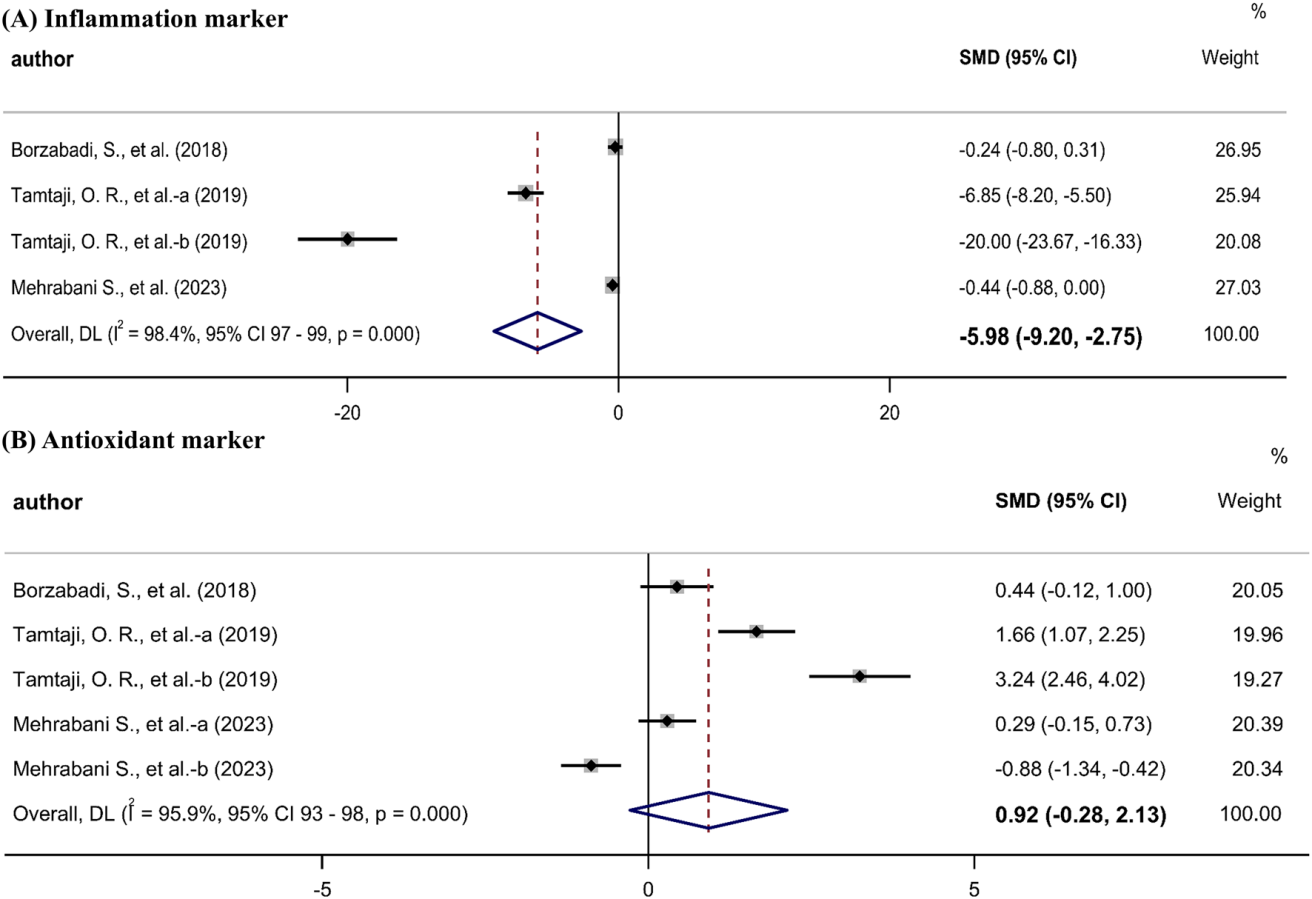
Meta-analysis of the effects of probiotics on inflammation in Parkinson’s disease.

Three studies [10, 14, 26] assessed serum inflammatory markers such as nitric oxide (μmol/L), malondialdehyde (mmol/L), and high-sensitivity C-reactive protein. The meta-analysis revealed a significant reduction in the serum levels of inflammatory markers in the probiotic groups (SMD, − 5.98; 95% CI − 9.20 to − 2.75) (Fig. 4A). The quality of evidence for inflammatory markers was estimated to be very low according to the GRADE system (based on inconsistency, indirectness, and publication bias) (Table 2).

Three studies [10, 14, 26] assessed total glutathione (μmol/L) and total antioxidant capacity (mmol/L) levels. The meta-analysis showed no significant difference (SMD, 0.92; 95% CI − 0.28 to 2.13) in antioxidant markers (Fig. 4B). The quality of the antioxidant marker data was estimated to be very low according the GRADE system (based on inconsistency, indirectness, and imprecision) (Table 2).

### Effect of probiotics on metabolic syndrome risk

**Fig. 5:**
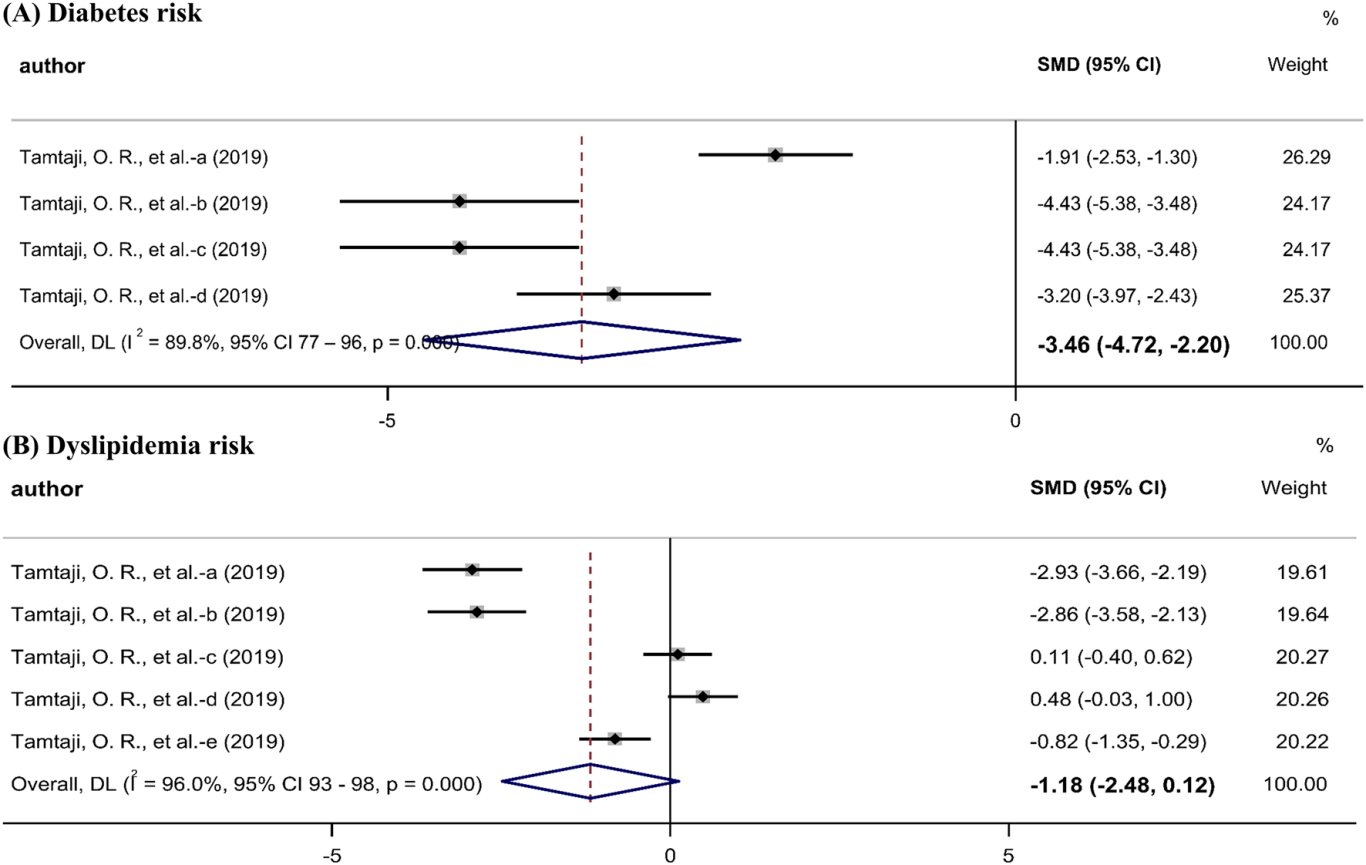
Meta-analysis of the effects of probiotics on metabolic syndrome risk in Parkinson’s disease.

Metabolic syndrome risk was identified by assessing the risk of diabetes and dyslipidemia. One study [14] included four comparisons pertaining to the risk of diabetes (fasting plasma glucose [mg/dL], serum insulin [mIU/mL], homeostasis model of assessment-estimated insulin resistance, and quantitative insulin sensitivity check index). The meta-analysis revealed a significant reduction in the risk of diabetes in the probiotic groups (SMD, − 3.46; 95% CI − 4.72 to − 2.20) (Fig. 5A). The quality of evidence for the risk of diabetes was estimated to be very low according to the GRADE system (based on inconsistency, indirectness, and publication bias) (Table 2).

The aforementioned study above [14] also included five comparisons of the risk of dyslipidemia (serum levels of triglycerides [mg/dL], very-low-density lipoprotein (VLDL)-cholesterol [mg/dL], total cholesterol [mg/dL], low-density lipoprotein (LDL)-cholesterol [mg/dL], and HDL-cholesterol [mg/dL]). There were no significant differences between the groups (SMD, − 1.18; 95% CI − 2.48 0.12) in the risk of dyslipidemia (Fig. 5B). The quality of evidence for the risk of dyslipidemia was estimated to be very low according to the GRADE system (based on inconsistency, indirectness, imprecision, and publication bias) (Table 2).

### Effects of probiotics on PD scale scores

**Fig. 6:**
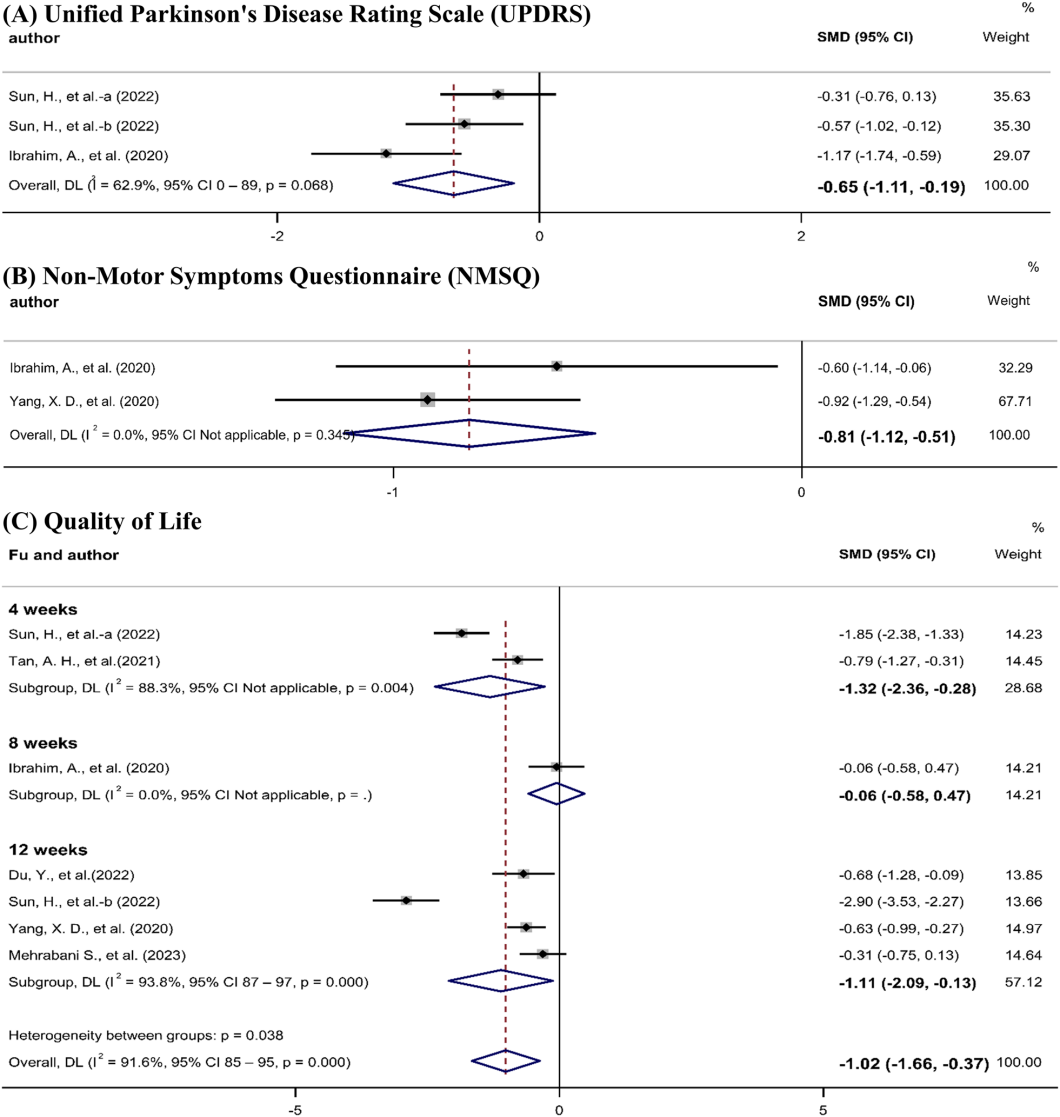
Meta-analysis of the effects of probiotics on Parkinson’s disease-related scales.

Two studies [12, 24] included UPDRS Part III motor scores, and there were significant improvements in the probiotic groups (SMD, − 0.65; 95% CI − 1.11 to − 0.19) (Fig. 6A). The UPDRS Part III quality of evidence was estimated to be high according to the GRADE system (Table 2).

Two studies [12, 16] reported Non-Motor Symptoms Questionnaire (NMSQ) scores. The meta-analysis revealed a significant reduction in NMSQ scores in the probiotic groups (SMD, − 0.81; 95% CI − 1.12 to − 0.51) (Fig. 6B). The NMSQ quality of evidence was estimated to be high according to the GRADE system (Table 2).

Quality of life was reported in six studies [12, 15, 16, 15–16] that included seven comparisons. There were significant improvements in the probiotic groups (SMD, − 1.02; 95% CI − 1.66 to − 0.37). Two studies [15, 24] that followed up after four weeks showed significant improvements in the probiotic groups (SMD, − 1.32; 95% CI − 2.36 to − 0.28). Four studies [16, 24–26] that followed up after 12 weeks also showed significant improvements in the probiotic groups (SMD, − 1.11; 95% CI − 2.09 to − 0.13) (Fig. 6C). The quality of evidence for quality of life was estimated to be moderate according to the GRADE system (this was reduced due to inconsistency) (Table 2).

### Effects of probiotics on patients' mental health

**Fig. 7:**
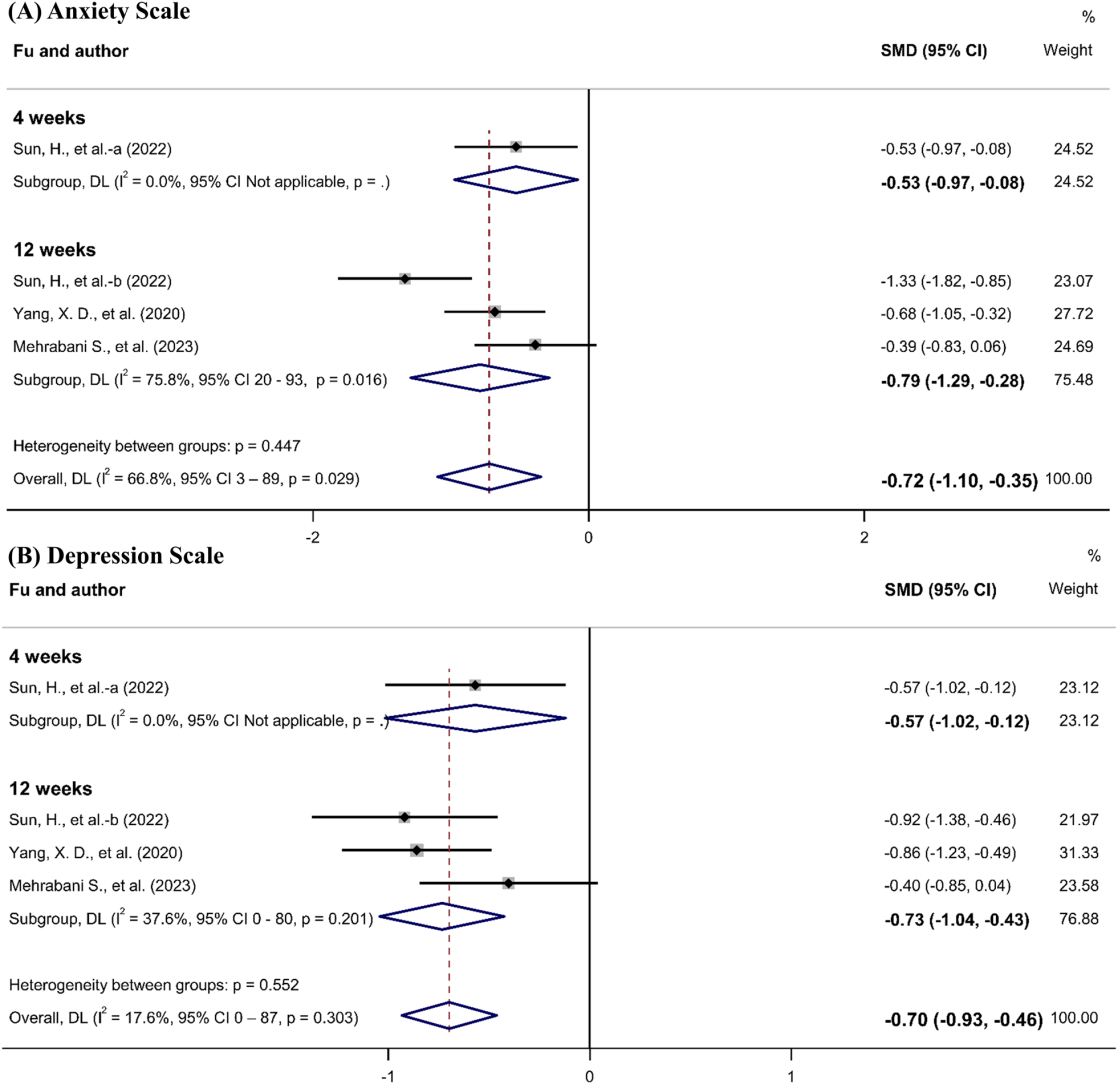
Meta-analysis of the effects of probiotics on mental health in Parkinson’s disease. Anxiety Scale.

Two studies [16, 24] used the Hamilton Anxiety Rating Scale (HAMA) and one study [26] used the Hospital Anxiety and Depression Scale (HADS) Anxiety subscale. There were significant improvements in the probiotic groups (SMD, − 0.72; 95% CI − 1.10 to − 0.35). In addition, three studies [16, 24, 26] that followed up after 12 weeks showed significant improvements in the probiotic groups (SMD, − 0.79; 95% CI − 1.29 to − 0.28) (Fig. 7A). The quality of evidence for the anxiety scales was estimated to be moderate according to the GRADE system (reduced due to inconsistency) (Table 2).

Two studies [16, 24] used the Hamilton Depression Rating Scale (HAMD) and one study [26] used the Hospital Anxiety and Depression Scale (HADS) Depression subscale. There were significant improvements in the probiotic groups (SMD, − 0.70; 95% CI, − 1.04 to − 0.43). In addition, three studies [16, 24, 26] that followed up after 12 weeks showed significant improvements in the probiotic groups (SMD, − 0.73; 95% CI, − 1.04 to − 0.43) (Fig. 7B). The quality of evidence for the depression scales was estimated to be high according to the GRADE system (Table 2).

### Subgroup analysis

**Fig. 8:**
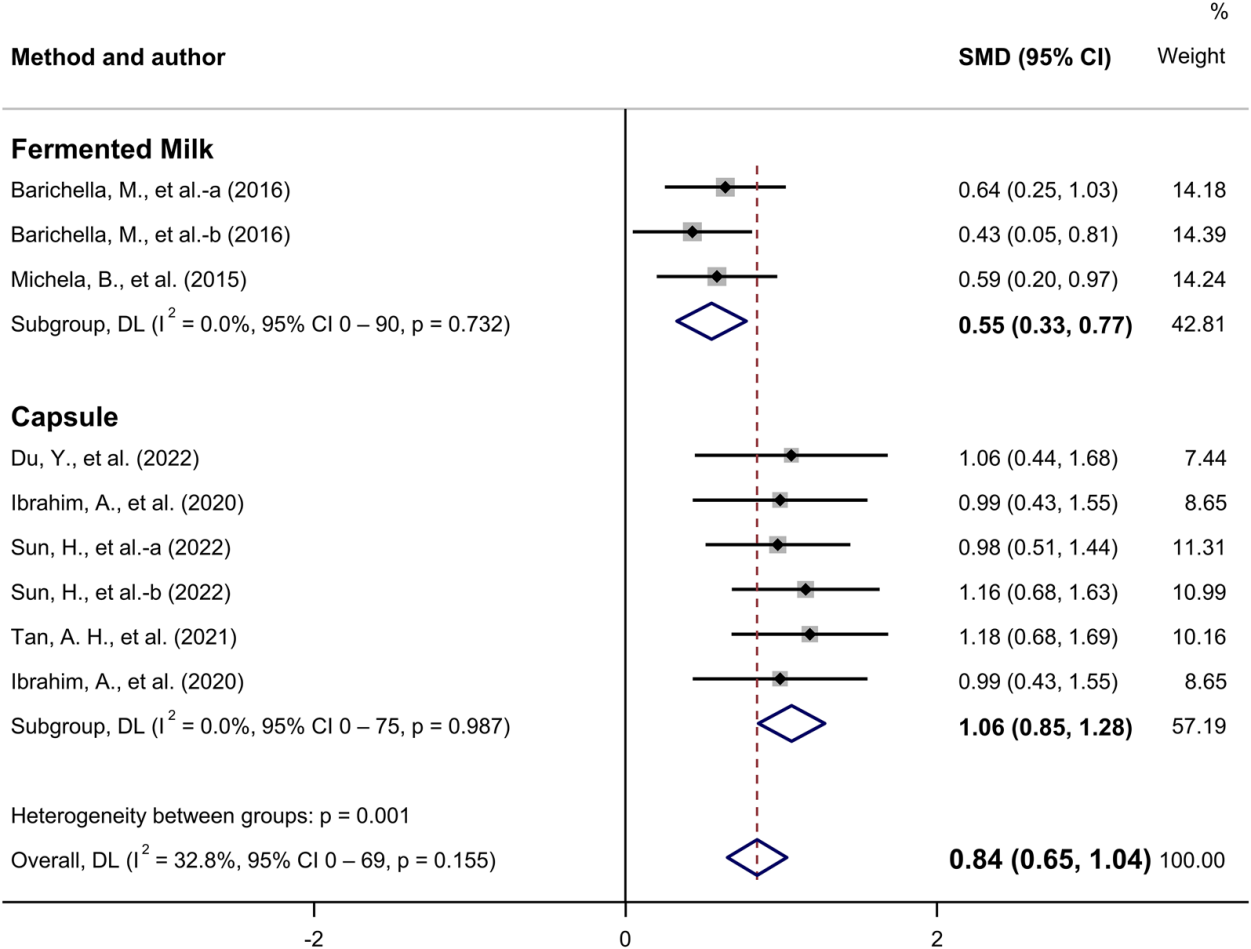
Subgroup analysis of the effects of probiotics on gastrointestinal motility by administration method

A subgroup analysis was performed according to the probiotic intake method. Both fermented milk (SMD, 0.55; 95% CI 0.33 to 0.77) and capsules (SMD, 1.06; 95% CI 0.85 to 1.28) demonstrated significant improvements in gastrointestinal motility (Fig. 8). The effect was greater when probiotics were ingested as capsules compared to when consumed as fermented milk. The quality of evidence was estimated as moderate according to the GRADE system (reduced due to publication bias) (Table 2).

### Safety/adverse events

Five [11, 14, 24–26] of the ten included studies reported no adverse effects. Two studies [9, 12] reported abdominal bloating, whereas one study [12] reported dizziness. Lethargy was reported in one patient, and this improved a week after discontinuation of the probiotics [15]. There were no reports of safety in any of the included studies, and no adverse events were reported in three studies [10, 13, 16].

## Discussion

This study performed a comprehensive and quantitative evaluation of randomized controlled trials on the effects of probiotics in patients with PD. We found high-quality evidence that probiotics significantly improve motor, non-motor PD symptoms, and depression. Moderate quality of evidence suggests that probiotics significantly increase gastrointestinal motility and quality of life and reduce anxiety. Low or very low quality evidence showed a significant reduction in serum levels of inflammatory markers and risk of diabetes. However, stool quality, constipation, antioxidant marker level, and risk of dyslipidemia were low or very low quality evidence, and there was no significant difference between the probiotic group and the control group. The improvement in gastrointestinal motility was greater when the probiotics were administered in capsules compared to when they were consumed in fermented milk forms. In addition, the longer the follow-up period, the better the gastrointestinal motility and mental health scores. These results suggest that probiotics can be considered as an adjuvant treatment option based on the pathophysiology of “gut dysbiosis” in patients with PD.

**Fig. 9:**
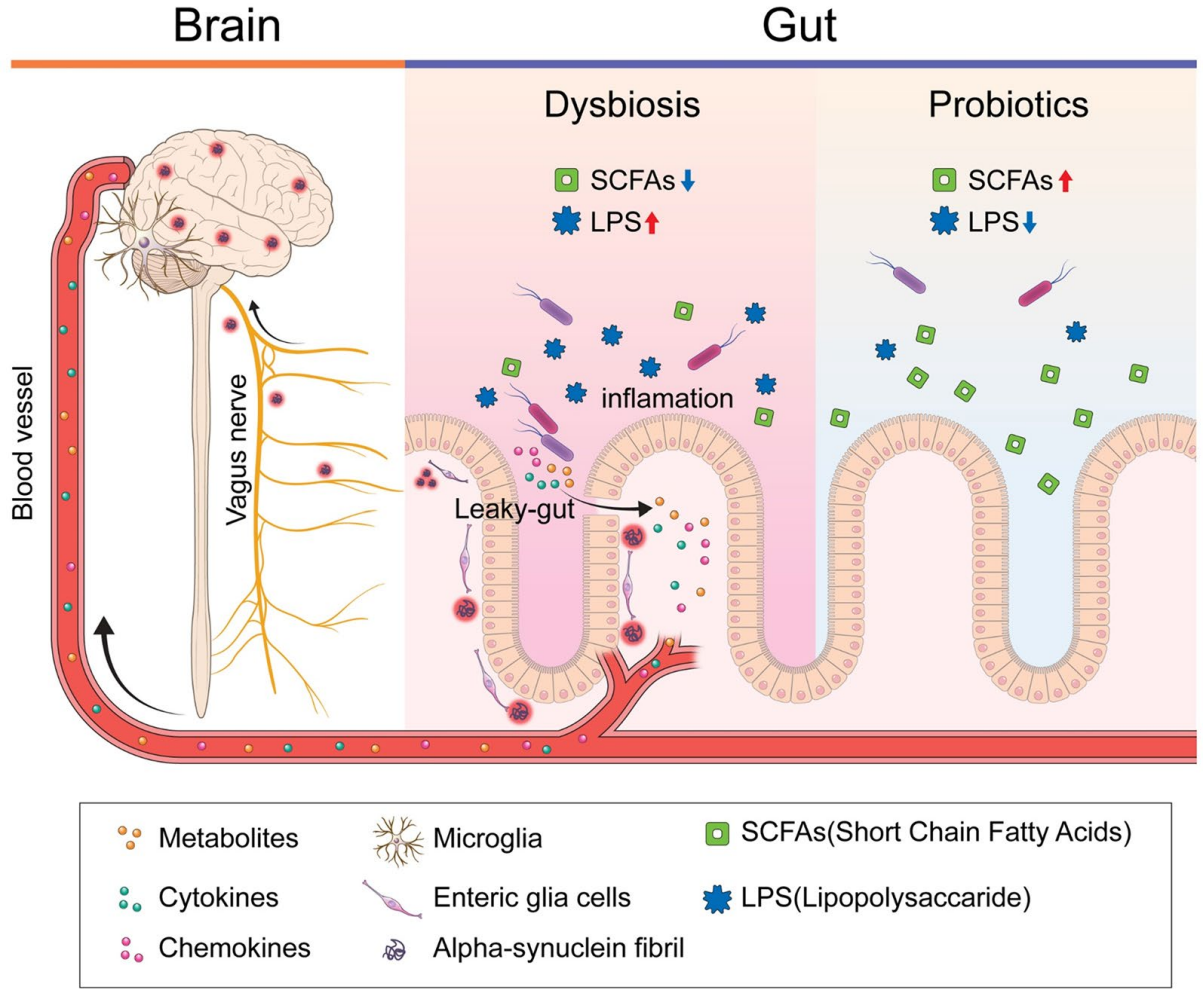
An overview of gut dysbiosis and the effects of probiotics in Parkinson’s disease. “Gut dysbiosis” decreases the levels of short-chain fatty acids (SCFAs) and increases the levels of lipopolysaccharides (LPS). In addition, it impairs intestinal epithelial barrier integrity (“leaky gut”) and triggers an inflammatory response, leading to the crossing of metabolites, chemokines, and cytokines through the intestinal wall into the bloodstream as well as through the blood–brain barrier. This inflammatory pathway also triggers misfolding of α-synuclein into enteric glial cells via the vagus nerve and into the brain. Probiotics alter the composition of microorganisms to increase SCFA levels, decrease LPS levels to reduce inflammation, and strengthen the intestinal barrier to prevent microbial migration. This figure was created using Medical Illustration & Design

. Studies from 1957 and 1960 revealed a loss of neurons in the substantia nigra and decreased dopamine levels in the striatum in patients with PD [27, 28]. In addition, degeneration of dopaminergic neurons in the substantia nigra compacta and projections to the striatum can induce PD. This denaturation appears as toxic aggregation of alpha-synuclein (α-syn), a major component of Lewy bodies [29]. Due to neuroinflammation through the blood–brain barrier and vagus nerve, α-syn, a pathological marker of PD, accumulates in the central nervous system, peripheral nervous system, and enteric nervous system [30, 31] (Fig. 9). Short-chain fatty acids (SCFAs) are major metabolites produced by microorganisms in the large intestine through anaerobic fermentation of undigested polysaccharides. SCFAs play an important role in maintaining intestinal barrier integrity, preventing microbial migration, and preventing inflammation by regulating the expression of tight junction proteins [32]. An imbalance in the gut flora due to increased harmful bacteria creates endotoxins (e.g., lipopolysaccharides [LPS]) and damages the intestinal barrier, thus triggering the migration of microorganisms and bacterial metabolites and consequently inducing pro-inflammatory pathways.

LPS interact with immune cells to induce cytokines, such as tumor necrosis factor and interleukins to induce systemic inflammatory responses [33]. LPS also interact with intestinal glial cells and brain microglia cells to activate inflammatory cytokines (nuclear factor kappa-light-chain-enhancer of activated B cells and pro-inflammatory cytokine interleukin-1β via cluster of differentiation 14 + and Toll-like receptor 4 receptors, which in turn produce neuroinflammatory α-syn aggregation in the vagus nerve and brain [34–38]. The vagus nerve directly innervates the myenteric plexus, where neurons run through the prevertebral ganglia within the spinal cord and finally to the brain. According to Braak’s hypothesis, pathogens enter the oral and nasal cavities and initiate the formation of Lewy bodies, resulting in sporadic PD [39]. Aggregation of α-syn initiates in the gut and olfactory sensory nerves, passes through the nasal olfactory lobe to the midbrain, and then the vagus nerve propagates α-syn to the brainstem and cortex [31, 40].

Bacteria that aid in SCFAs production include *Butyricicoccus*, *Clostridium *sensu stricto, *Roseburia*, and *Faecalibacterium prausnitzii*, whereas bacteria that decrease SCFAs and increase LPS include *Akkermansia, Escherichia/Shigella, Flavonifractor, Intestinimonas, Phascolarctobacterium,* and *Sporobacter* [41–47]. The effectiveness of probiotics on gut dysbiosis has been confirmed in several papers. In a *C. elegans* model of synucleinopathy, *Bacillus subtilis* was effective in eliminating α-syn aggregates and preventing their further aggregation [48]. In an in vitro model of the human colon microbiome, *Bifidobacterium longum subsp. infantis (B. infantis)* reduced the levels of LPS [49]. In addition, fermented milk containing lactic acid bacteria reduced LPS-induced neuroinflammation in a rat model [50]. Furthermore, in both in vitro and in vivo models of PD, probiotics reversed “gut dysbiosis” by altering the composition of the gut microbiome, thus disrupting pathways associated with inflammation [51, 52]. The above studies may explain the results that taking probiotics improves motor and non-motor symptoms in PD patients.

In addition to their effects on PD, the effects of probiotics on metabolic disorders are well known. The effects of probiotics on intestinal hormone and short-chain production affect glucose and lipid metabolism [53]. Supplementation with *Lactobacillus casei* has been shown to improve the glycemic response in diabetic patients by increasing levels of sirtuin 1, which is a key regulator of homeostasis and improves glucose metabolism by affecting gene expression. *Lactobacillus rhamnosus* has been reported to decrease blood glucose levels in diabetic mice by suppressing gluconeogenesis-associated gene expression, whereas *Lactobacillus acidophilus* has been shown to function as an antidiabetic agent by regulating the expression levels of genes related to glucose and lipid metabolism as well as inflammatory cytokines, such as glycogen synthase kinase 3β and sterol regulatory element-binding transcription factor 1c [54]. Certain strains of probiotics are capable of breaking down bile salts, resulting in a reduction in blood cholesterol levels. Probiotics also affect the expression of fasting-induced adipose factor (FIAF), which can limit the activity of lipoprotein lipase (LPL) and fat storage [55, 56]. However, unlike previous studies, this study found that taking probiotics reduced the risk of diabetes but did not make a significant difference to the risk of hyperlipidemia in PD patients. The evidence quality of this analysis is considered very low, most likely due to indirectness and imprecision resulting from merging markers from multiple blood tests from a single study.

## Strengths and limitations

The core strength of this meta-analysis is that we used quantitative statistical methods to determine the most comprehensive effects of probiotics in patients with PD.

Although there have been few clinical trials in humans, our meta-analysis provides insights into the effects of probiotics on PD. Additionally, we compared the effects of probiotics according to type and duration of administration.

This meta-analysis has several limitations. First, the number of studies and participants was small. To reduce heterogeneity between studies, we only included studies of participants diagnosed with idiopathic PD and excluded studies of patients with secondary parkinsonism, including a relatively small number of 840 patients. Second, we found significant heterogeneity among studies. This was confirmed not only through the I^2^ values but also through the 95% confidence interval [57]. We attempted to account for this heterogeneity by utilizing a random effects model and performing subgroup analyzes according to dosing method and follow-up period. However, heterogeneity remained high even after subgroup analysis was performed, suggesting additional sources of heterogeneity. In previous studies examining the different effects of probiotics reported that higher doses (≥ 10^10^ CFU), longer duration [58], and various strains of probiotics [59] were more effective. We therefore attribute the considerable heterogeneity of our study to the diversity of study protocols, such as probiotic strains, dosage and duration of intervention, and method of administration. These differences should be considered while interpreting the results. Third, the intervention durations of the included studies (ranging from 4 to 12 weeks) were short and insufficient to understand the long-term effects of probiotics. Fourth, some of the studies included in the meta-analysis may have been from the same center, which could have led to data overlap.

## Conclusions

Our study shows high-quality evidence that probiotics improve motor function, non-motor symptoms, and reduce depression in PD patients. Probiotic supplementation may be an affordable and safe adjuvant treatment option for PD management. To establish more trustworthy evidence on the potential benefits of probiotics for PD, it is necessary to conduct larger randomized controlled trials and long-term follow-up studies. The studies should be subdivided based on factors such as the severity of the disease, type and dosage of probiotics, duration of intervention, and they should include assessments of motor and cognitive function as well as other predictors of disease.

## Supplementary Information

**Additional file 1.** PRISMA 2020 checklist.

**Additional file 2.** Search date: 2023.02.20.

**Additional file 3.** Funnel plot to detect publication bias.

## Data Availability

The data and materials are available from the corresponding author upon request.

## Abbreviations

CI: Confidence interval
GRADE: Grading of recommendations, assessment, development, and evaluation
LPS: Lipopolysaccharides
MD: Mean differences
PD: Parkinson’s disease
SCFA: Short-chain fatty acid
SMD: Standard mean difference

## References

[1] Ou Z, Pan J, Tang S, Duan D, Yu D, Nong H (2021). Global trends in the incidence, prevalence, and years lived with disability of Parkinson's disease in 204 Countries/Territories From 1990 to 2019. Front Public Health.

[2] Borek LL, Amick MM, Friedman JH (2006). Non-motor aspects of Parkinson's disease. CNS Spectr.

[3] Skonieczna-Żydecka K, Marlicz W, Misera A, Koulaouzidis A, Łoniewski I (2018). Microbiome—the missing link in the gut-brain axis: focus on its role in gastrointestinal and mental health. J Clin Med.

[4] As AZS, Gogu AE, Chita DS, Frecus CE, Mihai CM (2022). Narrative review on therapeutic effects of dietary approach in neurological disorders. J Complement Med Res..

[5] Cox LM, Weiner HL (2018). Microbiota signaling pathways that influence neurologic disease. Neurotherapeutics.

[6] Li P, Killinger BA, Ensink E, Beddows I, Yilmaz A, Lubben N (2021). Gut microbiota dysbiosis is associated with elevated bile acids in Parkinson’s disease. Metabolites.

[7] Mosley RL, Hutter-Saunders JA, Stone DK, Gendelman HE (2012). Inflammation and adaptive immunity in Parkinson's disease. Cold Spring Harb Perspect Med.

[8] Morelli L, Capurso L (2012). FAO/WHO guidelines on probiotics: 10 years later. J Clin Gastroenterol.

[9] Barichella M, Pacchetti C, Bolliri C, Cassani E, Iorio L, Pusani C (2016). Probiotics and prebiotic fiber for constipation associated with Parkinson disease: an RCT. Neurology.

[10] Borzabadi S, Oryan S, Eidi A, Aghadavod E, Daneshvar Kakhaki R, Tamtaji OR (2018). The effects of probiotic supplementation on gene expression related to inflammation, insulin and lipid in patients with Parkinson's disease: a randomized, double-blind, PlaceboControlled Trial. Arch Iran Med.

[11] Georgescu D, Ancusa OE, Georgescu LA, Ionita I, Reisz D (2016). Nonmotor gastrointestinal disorders in older patients with Parkinson's disease: is there hope?. Clin Interv Aging.

[12] Ibrahim A, Ali RAR, Manaf MRA, Ahmad N, Tajurruddin FW, Qin WZ (2020). Multi-strain probiotics (Hexbio) containing MCP BCMC strains improved constipation and gut motility in Parkinson's disease: a randomised controlled trial. PLoS ONE.

[13] Michela B, Pacchetti C, Bolliri C, Cassani E, Iorio L, Pusani C (2015). Double blind, placebo-controlled trial of a fermented milk containing multiple probiotics strains and prebiotic fiber for constipation associated with parkinson’s disease. J Neurol Sci.

[14] Tamtaji OR, Taghizadeh M, Daneshvar Kakhaki R, Kouchaki E, Bahmani F, Borzabadi S (2019). Clinical and metabolic response to probiotic administration in people with Parkinson's disease: a randomized, double-blind, placebo-controlled trial. Clin Nutr.

[15] Tan AH, Lim SY, Chong KK, Manap MA, Hor JW, Lim JL (2021). Probiotics for constipation in Parkinson disease: a randomized placebo-controlled study. Neurology.

[16] Yang XD, He XQ, Xu SQ, Qian VV, Zhang Y, Song YY (2020). Effect of Lactobacillus casei supplementation on clinical response, gut microbiota and faecal metabolites in patients with Parkinson's disease a randomized, double-blind, placebo-controlled trial. Mov Disord.

[17] Lee SW, Koo MJ (2022). PRISMA 2020 statement and guidelines for systematic review and meta-analysis articles, and their underlying mathematics: life cycle committee recommendations. Life Cycle.

[18] Page MJ, McKenzie JE, Bossuyt PM, Boutron I, Hoffmann TC, Mulrow CD (2021). The PRISMA 2020 statement: an updated guideline for reporting systematic reviews. Syst Rev.

[19] Sterne JA, Savović J, Page MJ, Elbers RG, Blencowe NS, Boutron I (2019). RoB 2: a revised tool for assessing risk of bias in randomised trials. BMJ.

[20] Sterne JA, Egger M, Moher D. Addressing reporting biases. Cochrane handbook for systematic reviews of interventions: Cochrane book series. 2008:297–333.

[21] Fu R, Gartlehner G, Grant M, Shamliyan T, Sedrakyan A, Wilt TJ (2011). Conducting quantitative synthesis when comparing medical interventions: AHRQ and the effective health care program. J Clin Epidemiol.

[22] Guyatt GH, Oxman AD, Vist GE, Kunz R, Falck-Ytter Y, Alonso-Coello P (2008). GRADE: An emerging consensus on rating quality of evidence and strength of recommendations. BMJ.

[23] IntHout J, Ioannidis JP, Borm GF (2014). The Hartung-Knapp-Sidik-Jonkman method for random effects meta-analysis is straightforward and considerably outperforms the standard DerSimonian-Laird method. BMC Med Res Methodol.

[24] Sun H, Zhao F, Liu Y, Ma T, Jin H, Quan K (2022). Probiotics synergized with conventional regimen in managing Parkinson’s disease. NPJ Parkinsons Dis.

[25] Du Y, Li Y, Xu X, Li R, Zhang M, Cui Y (2022). Probiotics for constipation and gut microbiota in Parkinson’s disease. Parkinsonism Relat Disord.

[26] Mehrabani S, Khorvash F, Heidari Z, Tajabadi-Ebrahimi M, Amani R (2023). The effects of synbiotic supplementation on oxidative stress markers, mental status, and quality of life in patients with Parkinson’s disease: a double-blind, placebo-controlled, randomized controlled trial. J Funct Foods.

[27] Björklund A, Dunnett SB (2007). Dopamine neuron systems in the brain: an update. Trends Neurosci.

[28] Hornykiewicz O (2006). The discovery of dopamine deficiency in the parkinsonian brain. J Neural Transm Suppl.

[29] Burke WJ, Li SW, Williams EA, Nonneman R, Zahm DS (2003). 3,4-Dihydroxyphenylacetaldehyde is the toxic dopamine metabolite in vivo: implications for Parkinson’s disease pathogenesis. Brain Res.

[30] Kouli A, Torsney KM, Kuan WL, Stoker TB, Greenland JC (2018). Parkinson’s disease: etiology, neuropathology, and pathogenesis. Parkinson’s disease: pathogenesis and clinical aspects.

[31] Micieli G, Tosi P, Marcheselli S, Cavallini A (2003). Autonomic dysfunction in Parkinson's disease. Neurol Sci.

[32] Wang RX, Lee JS, Campbell EL, Colgan SP (2020). Microbiota-derived butyrate dynamically regulates intestinal homeostasis through regulation of actin-associated protein synaptopodin. Proc Natl Acad Sci.

[33] Ghosh SS, Wang J, Yannie PJ, Ghosh S (2020). Intestinal barrier dysfunction, LPS translocation, and disease development. J Endocr Soc.

[34] Letiembre M, Liu Y, Walter S, Hao W, Pfander T, Wrede A (2009). Screening of innate immune receptors in neurodegenerative diseases: a similar pattern. Neurobiol Aging.

[35] Rietdijk CD, Wezel RJ, Garssen J, Kraneveld AD (2016). Neuronal toll-like receptors and neuro-immunity in Parkinson’s disease, Alzheimer’s disease and stroke. Neuroimmunology and Neuroinflammation..

[36] Kim C, Ho DH, Suk JE, You S, Michael S, Kang J (2013). Neuron-released oligomeric α-synuclein is an endogenous agonist of TLR2 for paracrine activation of microglia. Nat Commun.

[37] Fellner L, Irschick R, Schanda K, Reindl M, Klimaschewski L, Poewe W (2013). Toll-like receptor 4 is required for α-synuclein dependent activation of microglia and astroglia. Glia.

[38] Fitzgerald E, Murphy S, Martinson HA (2019). Alpha-synuclein pathology and the role of the microbiota in Parkinson's disease. Front Neurosci.

[39] Rietdijk CD, Perez-Pardo P, Garssen J, van Wezel RJ, Kraneveld AD (2017). Exploring Braak's hypothesis of Parkinson's disease. Front Neurol.

[40] Braak H, Rüb U, Gai WP, Del Tredici K (2003). Idiopathic Parkinson's disease: possible routes by which vulnerable neuronal types may be subject to neuroinvasion by an unknown pathogen. J Neural Transm.

[41] Parada Venegas D, De la Fuente MK, Landskron G, González MJ, Quera R, Dijkstra G (2019). Short chain fatty acids (SCFAs)-mediated gut epithelial and immune regulation and its relevance for inflammatory bowel diseases. Front Immunol.

[42] Markello RD, Shafiei G, Tremblay C, Postuma RB, Dagher A, Misic B (2021). Multimodal phenotypic axes of Parkinson’s disease. Npj Parkinsons Dis..

[43] Forsyth CB, Shannon KM, Kordower JH, Voigt RM, Shaikh M, Jaglin JA (2011). Increased intestinal permeability correlates with sigmoid mucosa alpha-synuclein staining and endotoxin exposure markers in early Parkinson's disease. PLoS ONE.

[44] Rossi O, Khan MT, Schwarzer M, Hudcovic T, Srutkova D, Duncan SH (2015). *Faecalibacterium*
*prausnitzii* strain HTF-F and its extracellular polymeric matrix attenuate clinical parameters in DSS-induced colitis. PLoS ONE.

[45] Lenoir M, Martín R, Torres-Maravilla E, Chadi S, González-Dávila P, Sokol H (2020). Butyrate mediates anti-inflammatory effects of *Faecalibacterium*
*prausnitzii* in intestinal epithelial cells through Dact3. Gut Microbes.

[46] Yang B-G, Hur KY, Lee M-S (2017). Alterations in gut microbiota and immunity by dietary fat. Yonsei Med J.

[47] Yoon MY, Yoon SS (2018). Disruption of the gut ecosystem by antibiotics. Yonsei Med J.

[48] Goya ME, Xue F, Sampedro-Torres-Quevedo C, Arnaouteli S, Riquelme-Dominguez L, Romanowski A (2020). Probiotic *Bacillus*
*subtilis* protects against α-Synuclein aggregation in *C.*
*elegans*. Cell Reports..

[49] Engen PA, Green SJ, Voigt RM, Forsyth CB, Keshavarzian A (2015). The gastrointestinal microbiome: alcohol effects on the composition of intestinal microbiota. Alcohol Res.

[50] Rodes L, Khan A, Paul A, Coussa-Charley M, Marinescu D, Tomaro-Duchesneau C (2013). Effect of probiotics *Lactobacillus* and *Bifidobacterium* on gut-derived lipopolysaccharides and inflammatory cytokines: an in vitro study using a human colonic microbiota model. J Microbiol Biotechnol.

[51] Ghyselinck J, Verstrepen L, Moens F, Van Den Abbeele P, Bruggeman A, Said J (2021). Influence of probiotic bacteria on gut microbiota composition and gut wall function in an in-vitro model in patients with Parkinson's disease. Int J Pharm.

[52] Castelli V, d’Angelo M, Lombardi F, Alfonsetti M, Antonosante A, Catanesi M (2020). Effects of the probiotic formulation SLAB51 in in vitro and in vivo Parkinson's disease models. Aging.

[53] Musazadeh V, Roshanravan N, Dehghan P, Ahrabi SS (2022). Effect of probiotics on liver enzymes in patients with non-alcoholic fatty liver disease: an umbrella of systematic review and meta-analysis. Front Nutr.

[54] Zarezadeh M, Musazadeh V, Faghfouri AH, Sarmadi B, Jamilian P, Jamilian P (2022). Probiotic therapy, a novel and efficient adjuvant approach to improve glycemic status: an umbrella meta-analysis. Pharmacol Res.

[55] Musazadeh V, Zarezadeh M, Ghalichi F, Ahrabi SS, Jamilian P, Jamilian P (2022). Anti-obesity properties of probiotics; a considerable medical nutrition intervention: findings from an umbrella meta-analysis. Eur J Pharmacol.

[56] Zarezadeh M, Musazadeh V, Faghfouri AH, Roshanravan N, Dehghan P (2023). Probiotics act as a potent intervention in improving lipid profile: an umbrella systematic review and meta-analysis. Crit Rev Food Sci Nutr.

[57] Ioannidis JP, Patsopoulos NA, Evangelou E (2007). Uncertainty in heterogeneity estimates in meta-analyses. BMJ.

[58] Fernandez MA, Marette A (2018). Novel perspectives on fermented milks and cardiometabolic health with a focus on type 2 diabetes. Nutr Rev.

[59] Zhang Q, Wu Y, Fei X (2016). Effect of probiotics on glucose metabolism in patients with type 2 diabetes mellitus: a meta-analysis of randomized controlled trials. Medicina.

