# Supplementary material for: Effect of probiotic supplementation on gastrointestinal motility, inflammation, motor, non-motor symptoms and mental health in Parkinson’s disease: a meta-analysis of randomized controlled trials
Source: Gut Pathog. 2023 Mar 6;15:9. doi: 10.1186/s13099-023-00536-1 (PMC9990363; doi:10.1186/s13099-023-00536-1)
Supplement: Supplementary file 2 — Additional file 2. Search date: 2023.02.20. [file 13099_2023_536_MOESM2_ESM.docx]

**Additional file 2 Search date: 2023.02.20**

1. **Pubmed**

("Probiotics"[MeSH Terms] OR ("yeast s"[All Fields] OR "yeasts"[MeSH Terms] OR "yeasts"[All Fields] OR "yeast"[All Fields] OR "yeast, dried"[MeSH Terms] OR ("yeast"[All Fields] AND "dried"[All Fields]) OR "dried yeast"[All Fields] OR "saccharomyces cerevisiae"[MeSH Terms] OR ("saccharomyces"[All Fields] AND "cerevisiae"[All Fields]) OR "saccharomyces cerevisiae"[All Fields]) OR ("yoghurts"[All Fields] OR "yogurt"[MeSH Terms] OR "yogurt"[All Fields] OR "yoghurt"[All Fields] OR "yogurts"[All Fields]) OR "fermented product"[All Fields] OR "lactobacllus"[All Fields] OR ("bifidobacterium"[MeSH Terms] OR "bifidobacterium"[All Fields]) OR "fermented dairy product"[All Fields] OR ("synbiotics"[MeSH Terms] OR "synbiotics"[All Fields] OR "synbiotic"[All Fields]) OR "cultured milk products"[All Fields]) AND ("Parkinson Disease"[MeSH Terms] OR "Parkinsonian Disorders"[MeSH Terms] OR ("Parkinson Disease"[MeSH Terms] OR ("parkinson"[All Fields] AND "disease"[All Fields]) OR "Parkinson Disease"[All Fields] OR "parkinsons"[All Fields] OR "parkinson"[All Fields] OR "parkinson s"[All Fields] OR "Parkinsonian Disorders"[MeSH Terms] OR ("parkinsonian"[All Fields] AND "disorders"[All Fields]) OR "Parkinsonian Disorders"[All Fields] OR "parkinsonism"[All Fields] OR "parkinsonisms"[All Fields] OR "parkinsons s"[All Fields]) OR ("Parkinson Disease"[MeSH Terms] OR ("parkinson"[All Fields] AND "disease"[All Fields]) OR "Parkinson Disease"[All Fields] OR "parkinson s disease"[All Fields]) OR ("Parkinson Disease"[MeSH Terms] OR ("parkinson"[All Fields] AND "disease"[All Fields]) OR "Parkinson Disease"[All Fields] OR "parkinsons"[All Fields] OR "parkinson"[All Fields] OR "parkinson s"[All Fields] OR "Parkinsonian Disorders"[MeSH Terms] OR ("parkinsonian"[All Fields] AND "disorders"[All Fields]) OR "Parkinsonian Disorders"[All Fields] OR "parkinsonism"[All Fields] OR "parkinsonisms"[All Fields] OR "parkinsons s"[All Fields])) AND ("Prospective Studies"[MeSH Terms] OR "Cohort Studies"[MeSH Terms] OR "Randomized Controlled Trial"[Publication Type] OR ("Cohort Studies"[MeSH Terms] OR ("cohort"[All Fields] AND "studies"[All Fields]) OR "Cohort Studies"[All Fields] OR "cohort"[All Fields] OR "cohort s"[All Fields] OR "cohorte"[All Fields] OR "cohorts"[All Fields]) OR ("longitudinal studies"[MeSH Terms] OR ("longitudinal"[All Fields] AND "studies"[All Fields]) OR "longitudinal studies"[All Fields] OR "prospective"[All Fields] OR "prospectively"[All Fields]) OR "randomized cotrolled trial"[All Fields])

**(25 results)**

1. **EMBASE**

('probiotic agent'/exp OR probiotic OR yeast OR yogurt OR 'fermented product' OR lactobacillus OR bifidobacterium OR 'fermented dairy product' OR synbiotics OR 'cultured milk products') AND ('parkinson disease'/exp OR 'parkinsonism'/exp OR parkinson OR parkinsonism) AND 'randomized controlled trial'

**(54 results)**

1. **Cochrane**

ID Search Hits

#1 MeSH descriptor: [Probiotics] explode all trees 2635

#2 MeSH descriptor: [Parkinsonian Disorders] explode all trees 5067

#3 (#1 OR probiotic OR yeast OR yogurt OR fermented product OR lactobacillus OR bifidobacterium OR fermented dairy product OR synbiotics OR cultured milk products) AND (#2 OR Parkinson OR Parkinson’s disease OR parkinsonism) 52

**(52 results)**

1. **Scopus**

TITLE-ABS-KEY ( ( probiotic OR yeast OR yogurt OR "fermented product" OR lactobacillus OR bifidobacterium OR "fermented dairy product" OR synbiotics OR "cultured milk products" ) AND ( parkinson OR "Parkinson’s disease" OR parkinsonism ) AND ( "randomized controlled trial" OR "cohort" OR "prospective" ) )

**(78 results)**

1. **PsycINFO**

(probiotic OR yeast OR yogurt OR "fermented product" OR lactobacillus OR bifidobacterium OR "fermented dairy product" OR synbiotics OR "cultured milk products") AND (parkinson OR "Parkinson’s disease" OR parkinsonism)

**(69 results)**

1. **Web of Science**

(probiotic OR yeast OR yogurt OR "fermented product" OR lactobacillus OR bifidobacterium OR "fermented dairy product" OR synbiotics OR "cultured milk products") AND (parkinson OR "Parkinson’s disease" OR parkinsonism) AND 'randomized controlled trial'

**(15 results)**
