# Supplementary material for: Effect of probiotic supplementation on gastrointestinal motility, inflammation, motor, non-motor symptoms and mental health in Parkinson’s disease: a meta-analysis of randomized controlled trials
Source: Gut Pathog. 2023 Mar 6;15:9. doi: 10.1186/s13099-023-00536-1 (PMC9990363; doi:10.1186/s13099-023-00536-1)
Supplement: Supplementary file 3 — Additional file 3. Funnel plot to detect publication bias. [file 13099_2023_536_MOESM3_ESM.docx]

**Additional file 3. Funnel plot to detect publication bias**

|   (A) Gastrointestinal Motility |   (B) Stool Type- Bristol Stool Scale |
| --- | --- |
| ****  (C) Constipation symptom Reduction | ****  (D) Inflammation marker |
| ****  (E) Antioxidant marker | ****  (F) Diabetes risk |
|   (G) Dyslipidemia risk |   (H) Unified Parkinson's Disease Rating Scale (UPDRS)-Part III |
|   (I) Parkinson’s disease Questionnaire (PDQ-39) |   (J) Hamilton Anxiety Rating Scale(HAMA) |
|   (K) Hamilton Depression Rating Scale (HAMD) |   (L) Sub group analysis |
